# The production and biochemical characterization of α-carbonic anhydrase from *Lactobacillus rhamnosus* GG

**DOI:** 10.1007/s00253-022-11990-3

**Published:** 2022-05-25

**Authors:** Linda J. Urbański, Silvia Bua, Andrea Angeli, Reza Zolfaghari Emameh, Harlan R. Barker, Marianne Kuuslahti, Vesa P. Hytönen, Seppo Parkkila, Claudiu T. Supuran

**Affiliations:** 1 Faculty of Medicine and Health Technology, Tampere University, Arvo Ylpön katu 34, FI-33520 Tampere, Finland; 2 Sezione Di Chimica Farmaceutica E Nutraceutica, Neurofarba Department, Università Degli Studi Di Firenze, Via U. Schiff 6, I-50019 Sesto Fiorentino (Florence), Italy; 3 Department of Energy and Environmental Biotechnology, National Institute of Genetic Engineering and Biotechnology (NIGEB), 14965/161, Tehran, Iran; 4 Fimlab Ltd, Tampere University Hospital, Arvo Ylpön katu 4, FI-33520 Tampere, Finland

**Keywords:** Alpha carbonic anhydrase, *Lactobacillus rhamnosus*, Sulfonamide inhibition, Kinetics, In vitro inhibition

## Abstract

**Abstract:**

We report the production and biochemical characterization of an α-carbonic anhydrase (LrhCA) from gram-positive probiotic bacteria *Lactobacillus rhamnosus* GG. CAs form a family of metalloenzymes that catalyze hydration of CO_2_/interconversion between CO_2_ and water to bicarbonate ions and protons. They are divided into eight independent gene families (α, β, γ, δ, ζ, η, θ, and ι). Interestingly, many pathogens have been identified with only β- and/or γ-CAs, which can be targeted with CA-specific inhibitors (CAIs) acting as anti-pathogen drugs. Since it is important to study the potential off-target effects of CAIs for both the human body and its commensal bacteria, we took *L. rhamnosus* GG as our study subject. To date, only a single α-CA has been identified in *L. rhamnosus* GG, which was successfully produced and biochemically characterized. LrhCA showed moderate catalytic activity with the following kinetic parameters: k_cat_ of 9.86 × 10^5^ s^−1^ and k_cat_/K_M_ of 1.41 × 10^7^ s^−1^ M^−1^. Moderate inhibition was established with 11 of the 39 studied sulfonamides. The best inhibitors were 5-((4-aminophenyl)sulfonamido)-1,3,4-thiadiazole-2-sulfonamide, 4-(2-hydroxymethyl-4-nitrophenyl-sulfonamidoethyl)-benzenesulfonamide, and benzolamide with *K*_i_ values of 319 nM, 378 nM, and 387 nM, respectively. The other compounds showed weaker inhibitory effects. The *K*_i_ of acetazolamide, a classical CAI, was 733 nM. In vitro experiments with acetazolamide showed that it had no significant effect on cell growth in *L. rhamnosus* GG culture. Several sulfonamides, including acetazolamide, are in use as clinical drugs, making their inhibition data highly relevant to avoid any adverse off-target effects towards the human body and its probiotic organisms.

**Key points:**

• *The α-carbonic anhydrase from Lactobacillus rhamnosus GG (LrhCA) is 24.3 kDa.*

• *LrhCA has significant catalytic activity with a kcat of 9.9 × 105 s-1*.

• *Acetazolamide resulted in a marginal inhibitory effect on cell growth*.

## Introduction

To date, we know that there are similar numbers of cells from bacteria and other microorganisms (MOs) as there are individual cells in our body (Sender et al. 2016b); and when comparing nucleated cells, only the ratio reaches roughly 13:1 in favor of bacteria (Sender et al. 2016a). Many of our vital body functions depend on MOs living in our gut, mouth, stomach, skin, and urogenital areas including bacteria, archaea, eukarya, and even bacteriophages (Jalava 2008; Sender et al. 2016b). They affect human physiology, immunity, and nutrition, for example, by aiding us to food digestion, enabling vitamin intake, and reinforcing our immune system (Heikkilä 2018; Smith and Ravel 2017). Our body is constantly bombarded with numerous opportunistic pathogens. In many areas, much of our body’s natural defense system owes its efficiency to our commensal MOs. These microbes keep us healthy and our bodies functioning normally. The delicate balance of microflora can be easily disturbed through therapeutic applications available today, mainly antibiotics. Paradoxically, antibiotics are both beneficial and detrimental to the homeostasis of Mos in our body. They clear out many infectious agents while at the same time destroying protective commensal MOs. This is one of the reasons that there is an urgent need for medicines with novel mechanisms of action and an improved benefit/risk ratio.

Carbonic anhydrases (CAs) are ubiquitous metalloenzymes present in organisms spanning all kingdoms of life (Supuran and De Simone 2015). CAs belong to eight evolutionary divergent gene families (α, β, δ, γ, ζ, η, θ, and ι) that vary in tissue distribution, kinetics, inhibition, and activation profiles (Alterio et al. 2014; Frost and McKenna 2013; Jensen et al. 2019; Kikutani et al. 2016; Modak et al. 2013). These different classes share little amino acid or structural similarities but have convergent functionality. All of them require a metal ion (usually a zinc ion) at their active site, hence the name “metalloenzymes” (Kim et al. 2020). Their catalytic abilities transform CO_2_ via hydrolysis to bicarbonate and protons — a reaction that is essential for many vital physiological processes in organisms such as acid–base balance, gluconeogenesis, CO_2_ transport, and photosynthesis (Smith and Ferry 2000; Supuran 2011).

Excluding alpha CAs, other CA groups have emerged as new and promising anti-infective targets. This drives us towards designing CA-specific inhibitors (CAIs) that bind to the target CA and block its function, ultimately leading to the elimination of pathogens (Aspatwar et al. 2020; Kaur et al. 2020; Supuran et al. 2003). Current therapeutics, mainly antibiotics, have the unfortunate side effect of affecting not only target pathogens but also the probiotic bacteria in our bodies. In the same way, as the design of specific CAIs can reduce off-target effects against human CAs, it is equally important not to affect the CAs of commensal bacteria. From the eight known CA groups, humans, and some commensal lactobacilli — only possess CAs belonging to the α-group. Simultaneously, many pathogens have been discovered with only *β-* and/or *γ-CA*-encoding genes in their genomes (Supuran 2011). Based on this finding, CAIs designed to target β- and/or γ-CAs can be considered an intriguing option for antimicrobial drugs compared to classical antibiotics. By affecting only the CAs of target pathogens in the body, both the CA functions of human and the probiotic bacteria like lactobacilli will remain undisturbed.

*Lactobacillus* species are a normal part of the flora of human mouth, gastrointestinal system, and female genital tract (Bratcher 2018). In the latter, lactobacilli are known to produce lactic acid to create acidic conditions against pathogenic invasion (Witkin et al. 2007). In the gut and vagina, lactobacilli form biofilms that make them more resilient in harsh environmental conditions and maintain ample populations (Salas-Jara et al. 2016). Lactobacilli do not cause human disease, and some *Lactobacillus* species are used as consumable human probiotics (Barrons and Tassone 2008; Doron et al. 2005; Falagas et al. 2007; Reid 2001).

*Lactobacillus rhamnosus* is one example of probiotic bacteria which are used in dairy products, such as yogurt and semihard cheese. It was initially thought to be a subspecies of *L. casei* but was later confirmed as an individual species (Avlami et al. 2001). These short, gram-positive, facultative bacteria can grow either in the presence or absence of oxygen. *L. rhamnosus* has a broad spectrum of strains isolated from various environments such as the human gastrointestinal tract and vagina (Ceapa et al. 2016). *L. rhamnosus* GG is a strain isolated in 1983 from human intestinal tract by Sherwood Gorbach and Barry Goldin. Thereafter, *L. rhamnosus* GG has been studied extensively and is currently the world’s most studied probiotic bacterial species (Avlami et al. 2001; Silva et al. 1987).

In this study, we have produced and characterized a novel α-CA from *L. rhamnosus* GG. We also tested the inhibition properties of this enzyme using several sulfonamide compounds. Finally, we also investigated the effect of the clinical drug, acetazolamide, on the growth and survival of *L. rhamnosus* GG bacteria.

## Materials and methods

### Protein expression and recombinant production

**Fig. 1:**
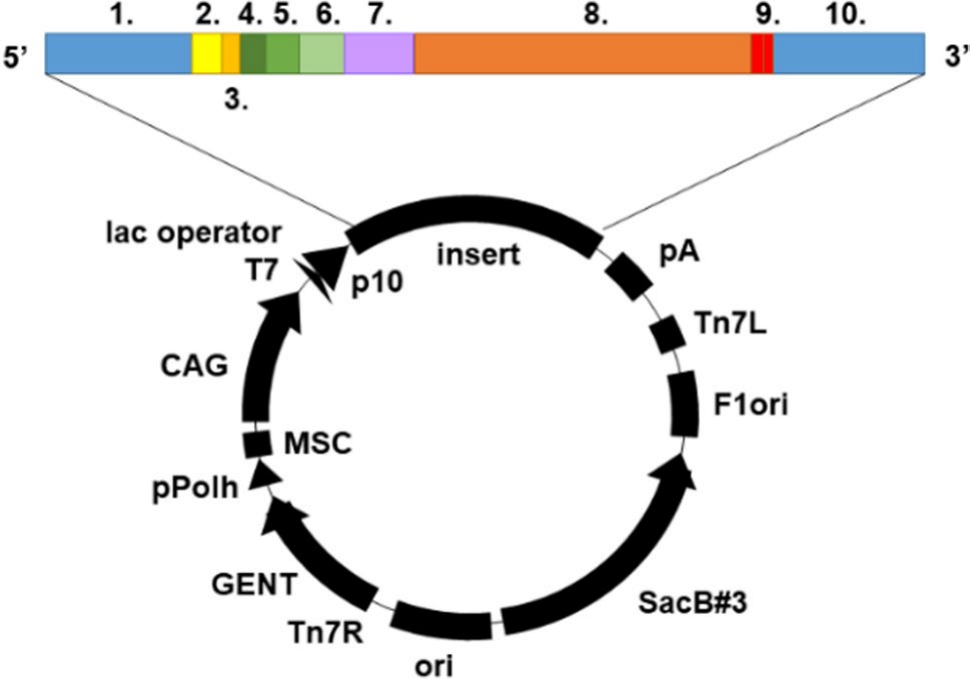
Illustration of the pBVboostFG expression vector used for recombinant production of the α-carbonic anhydrase from *L. rhamnosus* GG. The designed parts of the insert are as follows: (1) attL1, (2) Shine-Dalgarno, (3) Kozak, (4) Met-Ser-Tyr-Tyr, (5) 6xHis, (6) Asp-Tyr-Asp-Ile-Pro-Thr-Thr, (7) thrombin cleavage site (Lys-Val-Pro-Arg-Gly-Ser) (Hilvo et al. 2008), (8) CA gene of interest, (9) 2xstop codon, (10) attL2

We used a pBVboostFG-based plasmid (Laitinen et al. 2005) as protein expression vector to produce *L. rhamnosus* GG α-CA (UniProt (Hunter et al. 2009) protein entry: C8URX6) (Uniprot Consortium 2002-2017) in BL21 Star *Escherichia coli* cells (OneShot® BL21 Star™ (DE3) chemically competent cells, #C601003, Thermo Fisher Scientific, Waltham, US)A. The subcloned insert was designed to contain Gateway-compatible recombination sites (attL1, attL2), Shine-Dalgarno and Kozak sequences, 6xHis-tag with surrounding spacer regions (MSTT and ATAIPTT (Piao et al. 2008)), LrhCA, and a thrombin cleavage site (LVPRGS (Hilvo et al. 2008)) (Fig. 1).

GeneArt (Thermo Fisher Scientific, Waltham, USA) was responsible for gene synthesis and subcloning. Transformation — following the guidelines of Thermo Fisher Scientific OneShot® BL21(DE3) competent cells manual (part no. 28–0182). Cells were cultured at 37 °C in Luria–Bertani (LB) medium supplemented with 10 mg/mL gentamicin (1:1000, v/v) until optical density (OD_595_) of 0.4–0.6 was reached. Expression of the protein was induced by adding 1 M isopropyl β-D-1-isopropyl-thiogalactopyranoside (IPTG) 1:1000 (v/v), and afterwards, the culturing was continued overnight at 37 °C. Harvesting of the cells was performed by centrifugation at 5000 g for 15 min at 4 °C.

Harvested cells were mechanically disrupted with EmulsiFlex-C3 homogenizer (AVESTIN, Ottawa, Canada), and the lysate was centrifuged at 20,000 g for 15 min at 4 °C. Supernatant was combined with Protino® nickel-nitrilotriacetic acid (Ni^2+^-NTA) agarose affinity chromatography resin (Macherey–Nagel GmbH Co., Düren, Germany) and 50 mM Na_2_HPO_4_, 0.5 M NaCl, and 50 mM imidazole (binding buffer [BB]; pH 8.0, 1:3 [vol/vol]) for 2 h at room temperature (RT) on a constant agitation. After incubation, the resin was packed into chromatography column with an EMD Millipore™ vacuum filtering flask (Merck, #XX1004705, Darmstadt, Germany). Prior to elution, the resin was washed generously with BB. The protein was eluted with 50 mM Na_2_HPO_4_, 0.5 M NaCl, and 350 mM imidazole (pH 7.0). The eluted fractions were pooled and concentrated on a 5000 MWCO polyethersulfone membrane (Vivaspin 2, Vivascience Sartorius group, VS0211) and analyzed by sodium dodecyl sulfate–polyacrylamide gel electrophoresis (SDS-PAGE). The yield was determined by NanoDrop One (Thermo Fisher Scientific, Waltham, USA).

For further analysis and storage at 4 °C, the buffer of the protein sample was changed to 50 mM Tris–HCl (pH 7.5) with a 5000 MWCO polyethersulfone membrane (Vivaspin 2, Vivascience Sartorius group, VS0211). His-tag was removed from the purified protein with thrombin (Sigma-Aldrich, #RECOMT, St. Louis, USA) treatment according to the Sigma-Aldrich CleanCleave™ Kit manual (available on the Sigma-Aldrich website). Prior to use, the thrombin resin (Sigma-Aldrich, #RECOMT, St. Louis, MO, USA) was washed with 50 mM Tris–HCl (pH 7.5) according to the Sigma-Aldrich CleanCleave™ Kit manual. The protein sample was combined with the thrombin resin (1 ml of thrombin resin aliquot/1 mg protein) was incubated with the resin overnight at room temperature in gentle agitation. After incubation, the thrombin resin was packed into a chromatography column provided by the Sigma-Aldrich CleanCleave™ Kit, and the protein sample was collected. Analysis of cleavage reaction was done by SDS-PAGE.

### Kinetic studies

For assaying of CA-catalyzed CO_2_ hydration activity an Applied Photophysics, stopped-flow instrument was used (Khalifah 1971). The pH indicator was phenol red (at a concentration of 0.2 mM), working at the absorbance maximum of 557 nm, with 20 mM Hepes (pH 7.5) as buffer and 20 mM Na_2_SO_4_ (for maintaining constant ionic strength), following the initial rates of the CA-catalyzed CO_2_ hydration reaction for a period of 10 − 100 s. For the determination of the kinetic parameters and inhibition constants, CO_2_ concentrations ranged from 1.7 to 17 mM.

### Inhibitory studies

#### Sulfonamide inhibition

Inhibition of LrhCA was investigated with a set of designated sulfonamides, involving 15 clinically used drugs acetazolamide through hydrochlorothiazide (AAZ-HCT) and their heterocyclic derivatives (1–24**)**. For the determination of the initial velocity, at least six traces of the initial 5–10% of the reaction were used for each inhibitor. The uncatalyzed rates were determined similarly and subtracted from the total observed rates. Stock solutions of the inhibitor (0.1 mM) were prepared in distilled-deionized water. Subsequent series of dilutions up to 0.01 nM were prepared with distilled-deionized water. Inhibitor (I) and enzyme (E) solutions were preincubated together for 15 min at room temperature prior to the assay to allow formation of the E-I complex. The inhibition constants were obtained by nonlinear least squares methods using PRISM 3. The mean values were calculated from at least three different determinations (data not shown).

#### In vitro inhibition assay with acetazolamide

The effect of AAZ on cell growth was investigated with 96- and 24-well microplates (Nunc™, Thermo Fisher, Waltham, USA) and 15 ml test tubes. The plates and the test tubes were inoculated with (*OD*_595_ = 0.45) overnight anaerobic MRS pre-culture (37 °C, 170 rpm) of *L. rhamnosus* GG. The AAZ solution was prepared by diluting AAZ powder (Diamox by Mercury Pharmaceuticals Ltd., Fimlab, Tampere, Finland) with sterile water. Subsequently, the studied concentrations were done by serial dilution in MRS. In case of microplates, 4–6 separate samples were prepared with half of the well volume being cells and the other half of the studied AAZ dilution (1 nM, 10 nM, 100 nM, 1 µM, 10 µM, 100 µM, 1 mM, and 10 mM). Control samples without inhibitor (cells only) were prepared as well. The microplates and the test tubes were cultured anaerobically/aerobically in MRS at 37 °C and 230–400 rpm (PST-60HL Plate-Shaker-Thermostat, Biosan, Riga, Latvia/MaxQ™ 6000 Incubated Shaker, Thermo Fisher, Waltham, USA). The total culturing time was set for 96 h, with OD_595_ measurements in time points 0, 24, 48, 72, and 96 h. The cultures were analyzed for OD_595_ with UV/VIS Envision Multimode Plate Reader (PerkinElmer Oy, Finland). Each condition was studied three times, from which the mean was calculated to obtain the growth rate.

## Results

### Attempts of endogenous protein purification

The sequence data of the genome of *L. rhamnosus* (strain ATCC 53,103/GG) was obtained from UniProt and used to identify LrhCA gene (UniProt ID C8URX6) (Uniprot Consortium 2002-2017). The presence of the LrhCA gene in *L. rhamnosus* bacteria was confirmed using colony- and cDNA-polymerase chain reaction (PCR) (data not shown). To obtain the cDNA-PCR result, mRNA was isolated from the *L. rhamnosus* GG cells and transcribed to cDNA to see the expression level of the α-CA. Primers used in the studies are presented in Table 1.

**Table 1:** Primers for *L. rhamnosus* CA used in colony and cDNA-PCR

Primers	Sequences	Product (bp)
Forward	5′-ATGAACATGGCAGTTTTAGATTAT-3′	24
Reverse	3′-TTAGTTGGCAGTTTTGGTA-5′	19

Since *L. rhamnosus* could be obtained from commercial probiotic products (Gefilus Basic capsules containing *L. rhamnosus* GG strain, Yliopiston Apteekki, Helsinki, Finland), we first planned experiments to isolate CA protein from these bacteria. Attempts to purify LrhCA from the bacteria were designed according to the inhibitor affinity chromatography and procedures that have been previously used to isolate human endogenous CAs (Falkbring et al. 1972; Johansen 1976; Maupin et al. 2009; Wistrand and Knuuttila 1989). *L. rhamnosus* was successfully cultured on Petri dishes as well as in broth media (MRS, whey). After the inhibitor affinity chromatography, only a faint band equal to the theoretical size of LrhCA (*M*_w_ = 24.3 kDa) could be detected on SDS-PAGE. However, the identification of the protein by mass spectrometry (MS/MS) failed to confirm the product as LrhCA (data not shown). The purification procedure was repeated multiple times by culturing the cells in the presence or absence of O_2_ and by altering temperatures, pH, culturing time, medium broth (MRS-glucose, MRS-galactose, whey), the amount of resin, incubation time with the resin, and washing and elution procedures. In addition to *L. rhamnosus* GG, the cultured lactobacilli strains finally included *L. rhamnosus* DSMZ, *L. helveticus* FMB Lh5, and *L. paracasei* LPC-S01. After several unsuccessful attempts, we initiated recombinant production instead of isolating the endogenous CA protein.

### Recombinant protein production

**Fig. 2:**
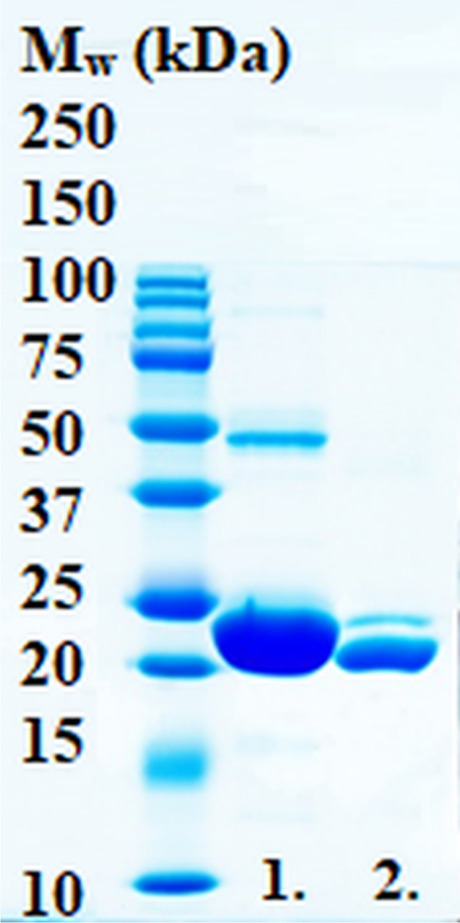
Coomassie-stained SDS-PAGE analysis of the produced recombinant α-CA of *L. rhamnosus* GG. The affinity-purified protein on lane 1 contains the 6xHis-tag, and a sample after thrombin cleavage and further purification is analyzed on lane 2, showing a trace of non-cleaved protein. On lane 1, the band marked as 1a presents the dimeric form of the protein, which is further seen as a fade band (marked as 2a) on lane 2 after His-tag removal. The amount of the protein is much larger on lane 1 compared to lane 2, which explains the more visible 1a band and the very faint 2a band. The standard molecular weight (M_w_) marker is illustrated on the far left. All polypeptide bands visible on the gel were identified as LrhCA by MS/MS

Recombinant LrhCA was successfully expressed in *E. coli* and purified by affinity chromatography. The yield of the protein was approximately 0.1 mg of purified protein per L of culture. Removal of the 6xHis-tag was carried out by treatment with thrombin (Sigma-Aldrich, St. Louis, MO, USA) followed by Ni^2+^-NTA purification and analysis via SDS-PAGE. In Fig. 2, the produced recombinant LrhCA is seen as a two-band polypeptide (lane 2, without His-tag). The size of the band visualized on the gel is in line with the theoretical mass of LrhCA, *M*_w_ = 24.3 kDa. All polypeptide bands on both lanes were identified as LrhCA by tandem mass spectrometry (MS/MS), the 48 kDa band therefore representing a dimer.

### Kinetic studies

The kinetic characteristics of LrhCA are shown in Table 2, alongside human α-CA isoforms I, II and III, for comparison.

**Table 2:** Kinetic properties of LrhCA, and human isoforms CA I, CA II, and CA III, for comparison

Enzyme	k_cat_ (s^−1^)	K_M_ (mM)	k_cat_/K_M_ (M^−1^ s^−1^)	K_i_ (AAZ^1^, nM)	Ref
LrhCA^5^	9.9 × 10^5^	7.0	1.4 × 10^7^	733	Current study
hCA I^2^	2.0 × 10^5^	4.0	5.0 × 10^7^	250	(Supuran 2008; Supuran et al. 2003)
hCA II^3^	1.4 × 10^6^	9.3	1.5 × 10^8^	12	(Supuran 2008; Supuran et al. 2003)
hCA III^4^	1.0 × 10^4^	33.3	3.0 × 10^5^	> 10,000	(Supuran 2008; Supuran et al. 2003)

^1^*AAZ* acetazolamide, ^*2*^*hCA I* human carbonic anhydrase isozyme I, ^*3*^*hCA II* human carbonic anhydrase isozyme II, ^*4*^*hCA III* human carbonic anhydrase isozyme III, ^*5*^*LrhCA* α-carbonic anhydrase of *Lactobacillus rhamnosus* GG

The kinetic results show that the enzymatic activity of LrhCA is between the values reported for human CA I and II. Notably LrhCA exhibited almost five times higher k_cat_ value compared to hCA I but 3.5 times lower k_cat_/K_M_.

### Inhibitory studies

#### Sulfonamide inhibition

**Fig. 3:**
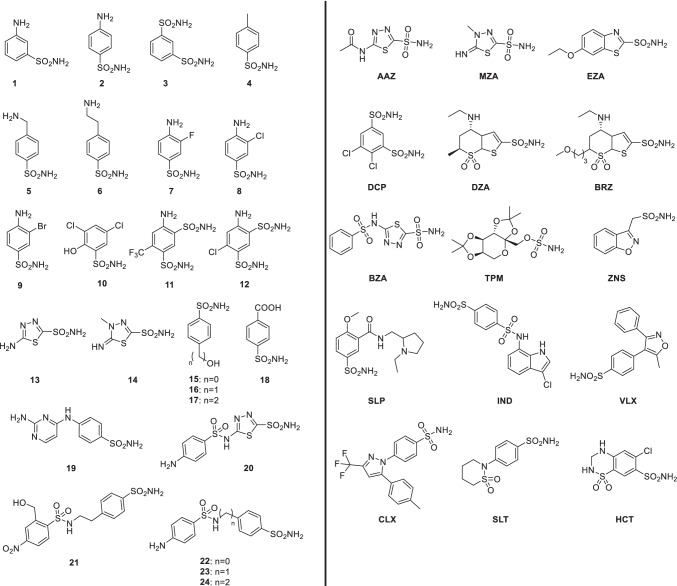
The molecular structures of sulfonamide derivates 1–24 and the clinically used sulfonamides AAZ-HCT examined in this study

Table 3 shows the inhibition results of designated sulfonamide derivatives 1–24 and the clinically used drugs AAZ-HCT. The molecular structures of AAZ-HCT and the derivatives 1–24 are shown in Fig. 3.

**Table 3:** Sulfonamide inhibition data of the α-carbonic anhydrase of *L. rhamnosus* (LrhCA) and human isoforms CA I and CA II, for comparison. All the CAs studied here belong to the enzyme class α

K_i_* (nM)			
Inhibitor	hCA Ia	hCA IIa	LrhCA
1	28,000	300	7611
2	25,000	240	7583
3	79	8	3019
4	78,500	320	5603
5	25,000	170	> 10,000
6	21,000	160	> 10,000
7	8300	60	> 10,000
8	9800	110	8983
9	6500	40	> 10,000
10	7300	54	5029
11	5800	63	7556
12	8400	75	7927
13	8600	60	866
14	9300	19	691
15	5500	80	> 10,000
16	9500	94	6006
17	21,000	125	681
18	164	46	2707
19	109	33	4407
20	6	2	319
21	69	11	378
22	164	46	559
23	109	33	487
24	95	30	403
AAZ	250	12	733
MZA	50	14	837
EZA	25	8	902
DZA	50,000	9	474
BRZ	45,000	3	433
BZA	15	9	387
TPM	250	10	508
ZNS	56	35	8946
SLP	1200	40	> 10,000
IND	31	15	> 10,000
VLX	54,000	43	> 10,000
CLX	50,000	21	> 10,000
SLT	374	9	9314
SAC	18,540	5959	> 10,000
HCT	328	290	> 10,000

^*^Mean from three different assays, measured by stopped-flow CO_2_ hydrase assay method (Khalifah 1971). Errors are in the range of 5–10% of the reported data. ^a^Human recombinant isozymes, data published previously (Supuran 2008)

From the inhibition data presented in Table 3, the following observations could be made:

(i) The three most efficient sulfonamide inhibitors for LrhCA were 5-((4-aminophenyl)sulfonamido)-1,3,4-thiadiazole-2-sulfonamide (compound 20), 4-(2-hydroxymethyl-4-nitrophenyl-sulfonamidoethyl)-benzenesulfonamide (compound 21), and benzolamide (BZA) with K_i_s of 319 nM, 378 nM, and 387 nM, respectively.

(ii) In 14 of the studied sulfonamides, the result was a *K*_i_ < 1 μM, 11 of which had a *K*_i_ of < 720 nM and could therefore be regarded as having a moderate inhibitory effect on LrhCA. These inhibitors were, in addition to those already mentioned in part (i), 4-(2-hydroxymethyl-4-nitrophenyl-sulfonamido)ethylbenzenesulfonamide (compound 24), brinzolamide (BRZ), dorzolamide (DRZ), 4-(4-sulfanilyl-aminomethyl)-benzenesulfonamide (compound 23), topiramate (TPM), sulfanylated sulfonamide (compound 22), 4-hydroxyethyl-benzenesulfonamide (compound 17), and 4-methyl-5-imino-1,3,4-thiadiazoline-2-sulfonamide (compound 14). The compounds with a K_i_ in the range of 733–902 nM and therefore considered to be medium-weak inhibitors were AAZ, methazolamide (MZA), 5-amino-1,3,4-thiadizole-2-sulfonamide (compound 13), and ethoxzolamide (EZA).

(iii) Out of the studied sulfonamides, 24 had a K_i_ value in the range of 1–10 μM, indicating little or no inhibition of LrhCA. These ineffective inhibitors were the simple derivatives 4-aminomethyl-benzenesulfonamides (compounds 5 and 6), 3-fluoro-4-amino-benzenesulfonamide (compound 7), 3-bromo-4-amino-benzenesulfonamide (compound 9), and 4-amino-/4-hydroxymethyl-benzenesulfonamide (compound 15) as well as the clinically used drugs sulpiride (SLP), indisulam (IND), valdecoxib (VLX), celecoxib (CLX), saccharin (SAC), and hydrochlorothiazide (HCT).

(iv) The clinically used drug AAZ showed moderate/weak inhibition of LrhCA with a *K*_i_ of 733 nM. The corresponding values with hCA I and hCA II are 250 nM and 12 nM, respectively.

#### Inhibitionin vitro with acetazolamide AAZ

Cultures of *L. rhamnosus* GG were prepared with varying concentrations of the classical inhibitor, AAZ (0 M, 1 nM, 10 nM, 100 nM, 1 µM, 10 µM, 100 µM, 1 mM, and 10 mM), to assess the effect of AAZ on cell growth. Lactobacilli were incubated both anaerobically and aerobically (different experiments) for a total of 96 h, with OD_595_ measurements at time points of 0, 24, 48, 72, and 96 h.

**Fig. 4:**
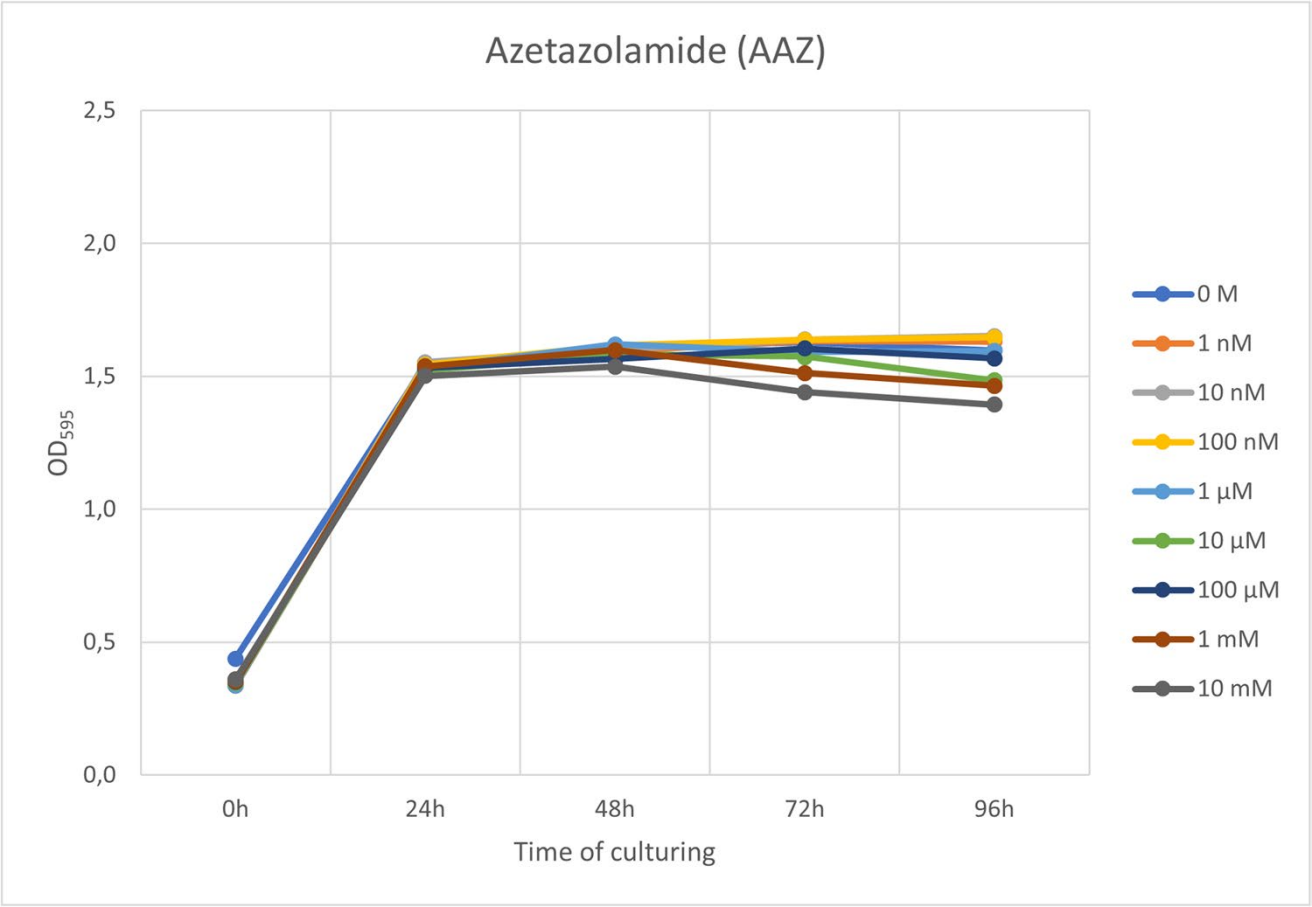
The effect of acetazolamide (in varying concentrations) on the growth of *L. rhamnosus* GG

It was discovered that AAZ had very little or no inhibitory effect on cell growth (Fig. 4). Most notable inhibitory effects could be seen with the highest concentrations (10 mM, 1 mM, and 100 µM) and after 48 h of culturing, but the differences in OD_595_ were still only marginal.

## Discussion

Numerous microorganisms residing inside the body greatly affect human health. These include archaea, bacteria, eukarya, and bacteriophages (Jalava 2008; Sender et al. 2016b). It is also clear that what we bring into our body alters the delicate balance of our microbiome. Antibiotics treat many bacterial infections, while they are also known to disturb the ecosystem of resident bacteria (Cully 2019). Around 1 in 10 people treated with antibiotics have digestive problems afterwards, and moreover, 1 in 15 people suffer from antibiotic-related allergies (NHS 2019). Such medications have been shown to have an effect on host physiology and health even 2 years after treatment (Tiessalo 2015). Another serious concern associated with the use of antibiotics is antibiotic resistance, caused by extensive use of drugs that is often unnecessary, excessive, or incomplete. This has risen to the level of a leading global challenge, with an estimated 700,000 yearly deaths attributed to drug-resistant diseases (French 2010). Understandably, novel medicines with different mechanisms of action are urgently needed to overcome, or at least minimize, the challenges of drug resistance. Recently, CAs have been introduced as potential new biomolecular drug targets in pathogenic infections. Several studies have shown that by interfering with CA activity, the growth and virulence of pathogens are impaired (Aspatwar et al. 2020, 2019; D'Ambrosio et al. 2018; De Vita et al. 2017; Pal et al. 2017, 2015). The design of such drugs, i.e., CAIs, stems from the findings that most pathogens have either β- or γ-CAs in their genomes, while, notably, these groups of CAs are absent in humans and lactobacilli. Many of the sulfonamides studied in this report are in use as clinical drugs making inhibition data important information when designing and using novel CAIs with the least adverse side/off-target effects towards human body and its probiotic organisms. Therefore, commensal and probiotic bacteria *L. rhamnosus* GG, which carries only an α-CA in its genome, is a fascinating study subject to learn if the use of CAIs targeting β- or γ-CAs is tolerated.

The α-CA of *L. rhamnosus* GG was successfully produced as a recombinant protein, resulting in a pure protein sample after metal-affinity chromatography. SDS-PAGE revealed a band corresponding to the theoretical *M*_w_ of 24.3 kDa and a trace of dimeric protein. A very similar result was reported previously by Li et al. (2015), whose production of α-CA from *L. delbrueckii* resulted in a double-band polypeptide on SDS-PAGE with the M_w_ of 23.8 kDa (Li et al. 2015). Prior to the recombinant protein production, we failed to isolate the endogenously produced CA from *L. rhamnosus* GG and from other *Lactobacillus* species tried. Inhibitory studies revealed that LrhCA has low affinity towards several sulfonamide CA inhibitors. Likely, this is the reason for the inhibitor affinity chromatography based on p-aminomethylbenzene sulfonamide not being successful. In a study by Mendes et al., another plausible explanation emerged; they observed no CA expression in *Lactobacillus plantarum* or in *L. delbrueckii* subsp. *bulgaricus* cultures supplied with CO_2_, even though the presence of the CA gene was confirmed (Mendes et al. 2013). It has also been discovered that CA expression is present only during non-exponential growth of bacteria, i.e., in stationary phases (16–31 h). This seems to occur when they shift from glucose fermentation to galactose and from homolactic to mixed acid fermentation (Laakso et al. 2011). Hence, we also attempted the isolation from lactobacilli cultured in the presence of both MRS-galactose instead of MRS-glucose and the same whey medium as used in the previous experiments. These experiments again showed no success. Although we consider the weak affinity of LrhCA towards the sulfonamide-coupled resin the most plausible explanation for the failure in isolation of the endogenous CA, the expression of α-CA in lactobacilli may also require very specific conditions, which warrant further studies.

A total of 11 of the 39 studied sulfonamides resulted in moderate inhibition; the rest had little or no inhibitory effect towards LrhCA. Many of these inhibitors possess a bulky scaffold with one or more substituents attached, which could interfere with effective binding within the active cavity of LrhCA. The most effective inhibitors were 5-((4-aminophenyl)sulfonamido)-1,3,4-thiadiazole-2-sulfonamide (compound 20), 4-(2-hydroxymethyl-4-nitrophenyl-sulfonamidoethyl)-benzenesulfonamide (compound 21), and BZA with K_i_ values of 319 nM, 378 nM, and 387 nM, respectively. Our inhibition study provides valuable information for the development of potent and more specific molecules against β- and γ-CAs which could be designed against various pathogenic infections. As it is important to avoid CAIs that have affinity towards the human α-CAs, it is also important to prevent similar effects on the α-CAs of commensal bacteria, such as lactobacilli. To gain more information on this topic, we also performed an in vitro study to see the effect of the clinically used drug, AAZ, on lactobacilli cell growth. AAZ showed no significant effect on the growth of *L. rhamnosus* GG. The relatively weak affinity of AAZ (K_i_ of 733 nM) towards LrhCA may be the main factor explaining this finding. It could also be possible that some of these bacteria express other CAs which compensate the potential loss of one enzyme. Of the known lactobacilli, *L. reuteri* has been identified with both an α-CA and additional γ-CA (Uniprot Consortium). However, we found no hits of a γ-CA or other CAs in the *L. rhamnosus* GG genome. The fact that AAZ did not inhibit the *L. rhamnosus* GG cell growth is a positive result considering the current usage of AAZ in treatment of glaucoma, epilepsy, etc. (Masini et al. 2013; Thiry et al. 2007). It is also encouraging for the future design of novel antimicrobial drugs resembling AAZ, since they may not impair the viability of the probiotic lactobacilli present in the human body.

## Data Availability

The authors confirm that the data supporting the findings of this study are available within the article.
